# Adult patent ductus arteriosus: An unusual cause of heart failure in an octogenarian female

**DOI:** 10.1007/s00508-016-1061-2

**Published:** 2016-08-26

**Authors:** Silvana Mueller, Fabian Plank, Katrin Klimes, Gudrun Feuchtner, Johannes Mair

**Affiliations:** 1 Department of Internal Medicine III – Cardiology and Angiology, Department of Pediatric Cardiology, Innsbruck Medical University, 6020 Innsbruck, Austria; 2 Department of Radiology, Innsbruck Medical University, 6020 Innsbruck, Austria; 3 Department of Internal Medicine III – Cardiology and Angiology, Innsbruck Medical University, Anichstrasse 35, 6020 Innsbruck, Austria

**Keywords:** Patent ductus arteriosus, Heart failure, Dyspnea, Octogenarians

## Abstract

Patent ductus arteriosus (PDA) is the third most common congenital abnormality in which the arterial duct, which normally closes spontaneously after birth within 24–48 h in full-term infants, remains permanently open. Breathlessness is very common in elderly patients and can be caused by several comorbidities, mostly cardiac and pulmonary diseases. PDA as a cause of heart failure in this patient population is very rare and diagnosis depends on high clinical awareness. Here we present a case diagnosed with multimodality imaging including 3‑dimensional (3D) transthoracic and transesophageal echocardiography and 3D-volume rendering technique (VRT) computed tomography.

## Introduction

A patent ductus arteriosus (PDA) is the third most common congenital abnormality in which the arterial duct, which normally closes spontaneously after birth within 24–48 h in full-term infants, remains permanently open [1]. Breathlessness is very common in elderly patients and can be caused by several comorbidities, mostly cardiac and pulmonary diseases. The PDA as a cause of heart failure in this patient population is very rare and diagnosis depends on high clinical awareness. In this article we present a case diagnosed with multimodality imaging including 3‑dimensional (3D) transthoracic and transesophageal echocardiography and 3D-volume rendering technique (VRT) computed tomography (CT).

## Case report

**Fig. 1:**
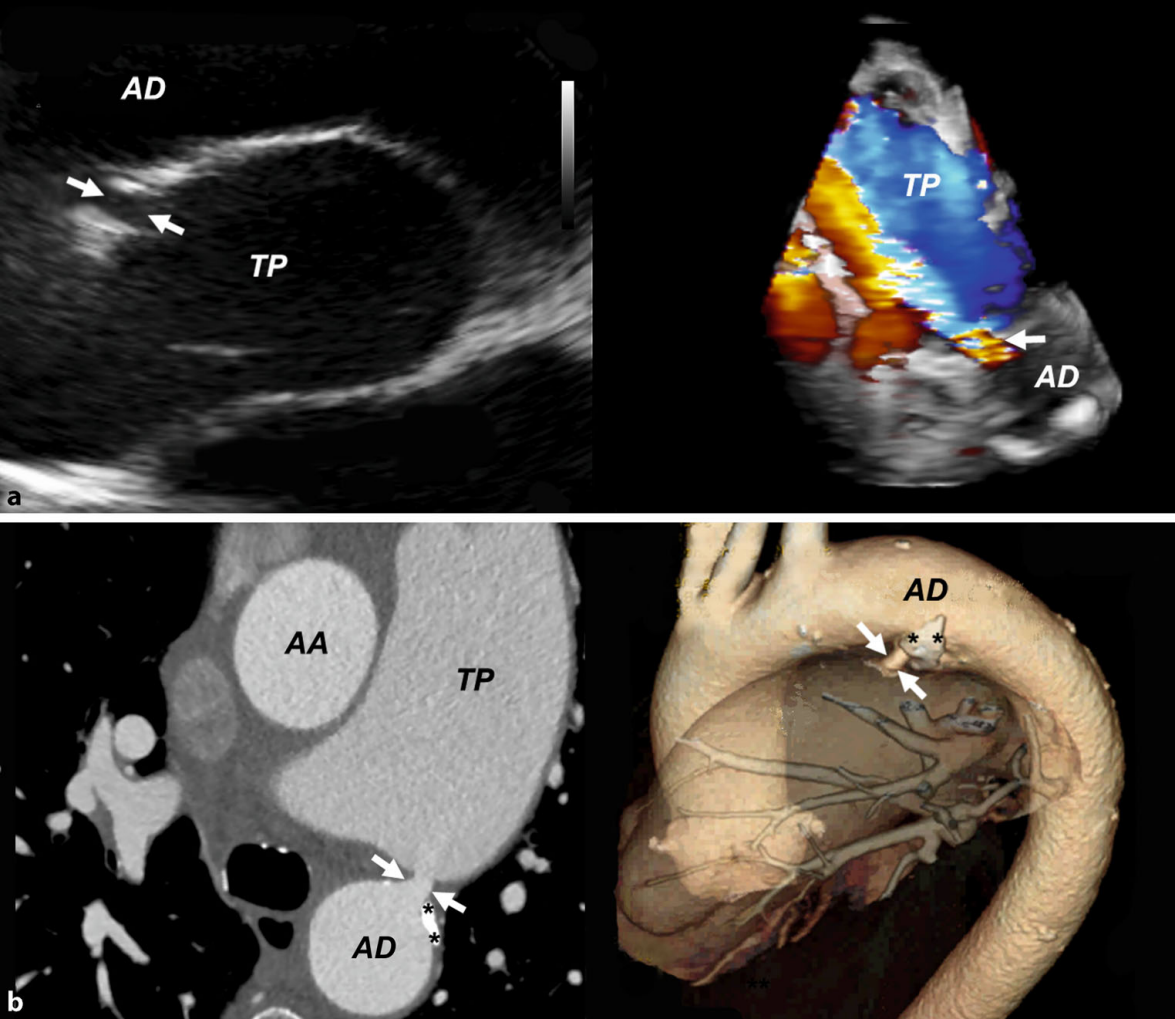
Multimodality imaging of patent partially calcified ductus arteriosus in an octogenarian female. *Upper panels* show images of transesophageal and 3‑D transthoraracic echocardiography, *lower panels* images of computed and 3D-VRT computed tomography. The diameter of the patent ductus arteriosus (*arrows*) was 6 mm, calcification is marked with asterisks (**). *AA* ascending aorta, *AD* descending aorta, *TP* pulmonary trunk

An 84-year-old female was referred for cardiac catheterization for evaluation of a suspected significant coronary artery disease and mitral valve regurgitation. The patient complained of worsening of dyspnea symptoms during exercise, New York Heart Association (NYHA) class II–III for several months without angina. The resting electrocardiogram (ECG) showed atrial fibrillation with a heart rate of 90/min (see supplemental figure 1). The N‑terminal pro B‑type natriuretic peptide (NT-proBNP) level was moderately elevated (2274 ng/l) with normal renal and thyroid function and borderline anemia (hemoglobin 116 g/l). Transthoracic and transesophageal 3D echocardiography (see Fig. 1, upper panels, supplemental video 1) revealed a rare cause of heart failure in octogenarians, i. e. a partially calcified but patent ductus arteriosus 6 mm in diameter, which was confirmed by 3D-VRT computed tomography (see Fig. 1, lower panels, supplemental video 2). All 4 heart chambers were dilated (echocardiography: left ventricular end-diastolic diameter 67 mm, right ventricle 35 mm, left atrium 59 mm and right atrium 48 mm), the pulmonary arteries (right 40 mm and left 30 mm) and the pulmonary trunk (55 mm) were severely dilated. Left ventricular systolic function was slightly decreased (left ventricular ejection fraction 45 %) and the right ventricular systolic function was normal. There was only mild to moderate mitral valve regurgitation and moderate pulmonary valve and tricuspid valve regurgitation. Cardiac catheterization revealed a significant left to right shunt of 0.56 (arterial, right atrial and pulmonary artery oxygen saturation was and 93 %, 27 % and 65 %, respectively) and severe pulmonary arterial hypertension (systolic 62 mm Hg and diastolic 28 mm Hg, mean 42 mm Hg, the pulmonary artery occlusion pressure was at maximum 14 mm Hg, mean 10 mm Hg; transpulmonary gradient 32 mmHg, diastolic pulmonary pressure gradient 18 mmHg). Significant coronary artery disease was ruled out by coronary angiography.

The subsequent detailed medical history revealed that the patient was first informed of the presence of a cardiac murmur at the age of 23 years and a holosystolic 3/6 systolic murmur (best heard right second intercostal space) could be heard at presentation to our department. At this young age the patient was asymptomatic but over the subsequent decades the substantial left to right shunt led to left ventricular dysfunction and severe pulmonary arterial hypertension.

## Discussion

It is very rare to diagnose a PDA in an octogenarian and the diagnosis requires a high clinical awareness as this congenital heart disease is uncommon in this patient population. The oldest published case was 91 years old [2] and patients can only occasionally survive over 50 years without an intervention [1–6] but our report confirms that PDA can be compatible with normal life expectancy. Systolic murmurs are unspecific as are the transthoracic echocardiographic signs as long as the PDA is not easily visible; however, a retrograde jet flow at the main pulmonary artery should raise the suspicion of PDA. In case of doubt in echocardiography, computed tomography enables definitive diagnosis.

Published data on the management of PDA in octogenarians is very limited [5, 6]. The relationship between the discovered PDA and the individual symptoms has to be assessed(incidental finding or relevant pathology?). In our case the significant left to right shunt is a plausible explanation for the development of left ventricular dysfunction, severe pulmonary hypertension and atrial fibrillation. A case of percutaneous occlusion of PDA with an Amplatzer duct occluder device in an 80-year-old man with moderate pulmonary hypertension has been reported [6]; however, transcatheter PDA closure may not be safe in patients with severe pulmonary hypertension and the PDA calcification seen in our patient even increases the interventional risk. Therefore, we started specific vasodilatory medication treatment of pulmonary arterial hypertension with an endothelin antagonist (macitentan 10 mg per day) during the hospital stay to lower the pulmonary pressure. At the 1‑year follow-up visit the patient presented with a good clinical condition, improved heart failure symptoms (NYHA II) and a significant decrease in NT-proBNP (808 ng/l). The transcutaneously measured oxygen saturation in the upper and lower limbs were 99 % each at rest and post-ductal 92 % in the lower limbs after a 6 min walking test (walking distance 420 m).

## Caption electronic supplementary material

- Fig. 1: Resting electrocardiogram at first presentation
- Supplemental video 1: Three-dimensional echocardiography of patent ductus arteriosus
- Supplemental video 2: 3D-VRT computed tomography of patent ductus arteriosus
